# The Spectrum Analysis Solution (SAS) System: Theoretical Analysis, Hardware Design and Implementation

**DOI:** 10.3390/s18020652

**Published:** 2018-02-22

**Authors:** Ram M. Narayanan, Richard K. Pooler, Anthony F. Martone, Kyle A. Gallagher, Kelly D. Sherbondy

**Affiliations:** 1 Department of Electrical Engineering, The Pennsylvania State University, University Park, PA 16802, USA; 2 RF and Millimeter-Wave Engineering Group, Johns Hopkins University Applied Physics Laboratory, Laurel, MD 20723, USA; rick.pooler@jhuapl.edu; 3 Sensors and Electronics Directorate, U.S. Army Research Laboratory, Adelphi, MD 20783, USA; anthony.f.martone.civ@mail.mil (A.F.M.); kyle.a.gallagher3.civ@mail.mil (K.A.G.); kelly.d.sherbondy.civ@mail.mil (K.D.S.)

**Keywords:** cognitive radar, dynamic spectral sharing (DSS), signal analyzer, spectral opportunity, spectral occupancy

## Abstract

This paper describes a multichannel super-heterodyne signal analyzer, called the Spectrum Analysis Solution (SAS), which performs multi-purpose spectrum sensing to support spectrally adaptive and cognitive radar applications. The SAS operates from ultrahigh frequency (UHF) to the S-band and features a wideband channel with eight narrowband channels. The wideband channel acts as a monitoring channel that can be used to tune the instantaneous band of the narrowband channels to areas of interest in the spectrum. The data collected from the SAS has been utilized to develop spectrum sensing algorithms for the budding field of spectrum sharing (SS) radar. Bandwidth (BW), average total power, percent occupancy (PO), signal-to-interference-plus-noise ratio (SINR), and power spectral entropy (PSE) have been examined as metrics for the characterization of the spectrum. These metrics are utilized to determine a contiguous optimal sub-band (OSB) for a SS radar transmission in a given spectrum for different modalities. Three OSB algorithms are presented and evaluated: the spectrum sensing multi objective (SS-MO), the spectrum sensing with brute force PSE (SS-BFE), and the spectrum sensing multi-objective with brute force PSE (SS-MO-BFE).

## 1. Introduction

In the United States, the Federal Communications Commission (FCC) regulates the radio frequency (RF) portion of the electromagnetic spectrum for non-federal use. The current FCC spectrum management policy allocates explicit blocks of the spectrum to specified applications with harsh interference penalties. This regulatory policy follows an exclusive-use model in which spectrum bands are licensed to applications for exclusive use [1]. Since 1994, the FCC has auctioned off large pieces of the spectrum to private industry in keeping with the high spectral demand. Allocating the spectrum in such a rigid manner has a lasting impact on the progress and capability of RF devices.

Over the past several years, there has been an exponentially increasing number of RF applications as well as an equally increasing demanded BW for these applications. For this reason, the RF spectrum is rapidly becoming an overcrowded, or congested, environment. Countless RF applications, such as mobile phones, have undergone explosive growth over the past two decades. Although the spectrum is becoming more congested and an increasing number of bands continue to be allotted for exclusive use, a study by the FCC Spectrum Policy Task Force shows that much of the spectrum remains idle [2]. A paradox lies in the fact that the spectrum is a highly sought-after commodity even though there is an abundance of unused spectrum. A widely accepted belief among the research community is that the current exclusive spectrum allocation model is not sustainable. The increasing demand for spectral capacity cannot continue to be met while using an RF spectrum allocation system that limits the spectral resources. Therefore, a more robust RF spectrum allocation system must be established. The inefficiency of the current exclusive-use spectrum allocation model necessitates a new RF paradigm that exploits the spectrum in a temporal and spatial sense.

One solution that has been presented by the cognitive radio community lies in allowing dynamic spectrum access (DSA), in which devices can co-share or co-exist in a sub-band of the spectrum [3]. By employing DSA techniques, devices are able to access the spectrum in a selection of unused channels. In a co-sharing scheme, the devices sharing a sub-band of the spectrum are in direct communication with one another, and can therefore agree upon a usage schedule. A co-existence scheme is trickier in the sense that the devices in the spectrum are not in direct communication. RF co-existence can occur in the fast-time, slow-time, spatial, and spectral domains, although this paper focuses primarily on the fast-time adaptation. These SS technologies use an understanding of the spectral environment to support adaptability to allow for more efficient spectral usage. A perception of the electromagnetic environment is therefore key in determining spectral opportunity. DSA has a broad meaning that encompasses several regulatory approaches to support SS.

A solution to mitigate spectrum shortage is to loosen spectrum regulation and allow for a cooperative approach to band sharing. Such opportunistic spectrum access (OSA), which may resolve the problem that is caused by the finite supply and increasing demand of usable spectrum by utilizing the idle portions of the spectrum. One infrastructure for spectrum management is the open sharing model (also known as the spectrum commons) that grants freedom for peers to share the spectrum as they see fit [4]. The open sharing model would require no regulation of the spectrum as access would be completely opportunistic and user managed. In an open sharing model, users are faced with the substantial task of developing new strategies for sharing the spectrum with an unlimited number of users without causing interference. In the dynamic exclusive model, bands are licensed for exclusive use to fixed applications [5]. The additional flexibility offered in the open sharing model stems from the fact that applications are granted exclusive use of the spectrum for a specific location and time as opposed to a static assignment in the traditional exclusive use model. The hierarchal model acts as a middle ground between the dynamic exclusive and the open sharing models. In the hierarchal model, adaptive RF networks are defined as visitors, or secondary users, in the band that they are accessing [6]. A hierarchal model allows for OSA without infringing on the rights of the primary user in the spectrum.

Recently, cognitive radar systems have been proposed and investigated for improved target detection capability [7,8,9]. The basic architecture of a cognitive radar consists of three elements: (1) intelligent signal processing, which builds on learning through interactions of the radar with the surrounding environment; (2) feedback from the receiver to the transmitter, which is a facilitator of intelligence; and, (3) preservation of the information content of radar returns, which is realized by the Bayesian approach to target detection through tracking [7]. In a cognitive radar, the transmitter adjusts the spectral, temporal, and spatial features of its illumination intelligently, taking into account the dynamically varying characteristics of the target and clutter response and radio frequency interference (RFI).

In order to ensure efficient and interference-free operation of cognitive radar to optimize target detection performance, the radar illuminator must dynamically sense and avoid spectral regions wherein RFI is present [10,11,12,13,14]. Based upon the detection of such interference bands or sub-bands, appropriate waveform design strategies can then be employed to enhance cognitive radar performance [15,16,17,18,19]. This paper significantly expands on an earlier version of our work published recently [20] and provides extensive details on the hardware, signal processing, and data analysis.

In this paper, we discuss a SS hardware architecture and associated signal processing designed to work in conjunction with and support of a cognitive radar requiring an active understanding of the RF environment. Section 2 presents an overview of the statistical measures that are used in characterizing the different sub-band selection approaches described later in Section 3. Hardware design and software implementation considerations of the Spectrum Analysis Solution (SAS) system are presented in Section 4. Section 5 presents data collection and data analysis. Finally, Section 6 presents conclusions and directions for future work in this area.

The novelty of our paper lies in the development, validation, and comparison of optimization algorithms for dynamic spectrum sensing to support the operation of a cognitive radar. These algorithms expand upon the existing spectrum sensing, multi-objective optimization model so that mutual interference between radar and communication systems is further mitigated. The successful application of these algorithms to the SS system, their real-time implementation, and the manner in which considerable flexibility is incorporated within to address different scenarios is also a novel contribution in this paper.

## 2. Spectrum Sensing Statistical Measures

**Figure 1:**
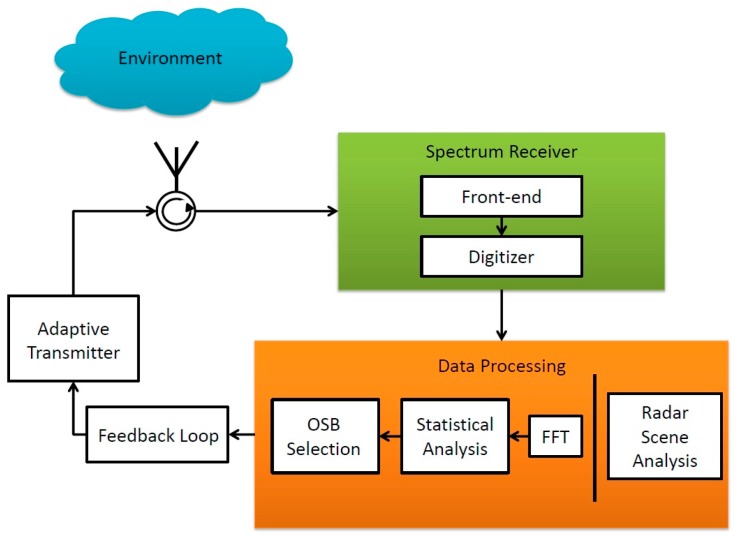
Block diagram of a typical spectrum sensing (SS) radar.

Figure 1 provides a general block diagram of a SS radar. This paper focuses on the spectrum receiver and data processing boxes in the SS radar block diagram. A spectrum receiver conditions and digitizes the RF energy in the environment. A fast Fourier transform (FFT) converts the discrete spectrum data from the time domain to the frequency domain. Next, a statistical analysis leads to the determination of an optimal OSB of transmission. An OSB is a portion of the spectrum and is an ideal band for radar transmission in its environment and mission profile for a given modality of operation [21]. Note the existence of feedback loop between the Data Processing block and the Adaptive Transmitter in Figure 1. The OSB is communicated to an adaptive transmitter that generally consists of one or more transmitting elements and an adaptive RF front-end. The adaptive transmitter transmits a waveform within the OSB. The transmitted signal is sent back to the processor for matched filtering which is performed in the Radar Scene Analysis block. The radar scene analysis is a statistical interpretation of a radar echo signal that provides information about the environment [7].

Consider a time domain signal x(t) over a time duration τ whose in-phase and quadrature components are denoted as $x_{I}(t)$ and $x_{Q}(t)$, respectively. Thus, we have

(1) $$x(t)=\sqrt{x_{I}^{2}(t)+x_{Q}^{2}(t)}.$$

If the signal is sampled at intervals of Δτ, the number of samples N is given by N=τ/Δt. If the sampled values of the signal and its in-phase and quadrature components are denoted, respectively, as x(n), $x_{I}(n)$ and $x_{Q}(n)$, for n=1,2,…,N, then we have

(2) $$x(n)=\sqrt{x_{I}^{2}(n)+x_{Q}^{2}(n)}.$$

After performing a K-point Fast Fourier Transform (FFT) operation, the power spectral density (PSD) is obtained as

(3) X(k)=F{x(n)}, k=1,2,…,K,

where k represents a frequency bin of the signal and K is the total number of frequency bins. The frequency bin size, or the resolution bandwidth (RBW) Δω in the PSD, is given by

(4) $$\Delta \omega =\frac{\omega_{K}-\omega_{0}}{K},$$

where $\omega_{0}$ is the start frequency and $\omega_{K}$ is the stop frequency.

The system noise power at the output of a receiver system arising from the noise signal $x_{n}(t)$, whose sampled values are denoted as $x_{n}(n)$, is computed using

(5) $$P_{n}=GF_{n}k_{B}TB,$$

where G is the receiver gain, $F_{n}$ is the receiver noise figure, $k_{B}$ is the Boltzmann constant, T is the temperature, and B is the receiver bandwidth. In a similar fashion as seen in Equation (3), the PSD of noise, $X_{n}(k)$, can be obtained. System noise plays a quintessential role in the interpretation of spectral data, particularly in the calculation of PO and SINR, as will be evident later.

### 2.1. Percent Occupancy (PO)

PO is a statistic frequently used in cognitive radio applications to characterize the spectral capacity [22,23,24]. PO is a point-by-point comparison of the power spectrum to a threshold, γ, which is commonly generated on the basis of a system’s noise floor. Note that PO is interchangeable with the term “spectrum occupancy”. PO is a two state process wherein the first state is labeled “occupied” and the complementary state is labeled “open” [25]. Consider a PSD X(k), as defined in Equation (3). We define $b_{k}$ as a binary parameter (either 0 or 1), which determines whether the *k*-th sample of X(k) is above a threshold, γ [26]. This binary parameter can be defined as

(6) $$b_{k}=\left\{ \begin{array}{lll} 0, & & \mathrm{if}\;X(k)<\gamma \\ 1, & & \mathrm{if}\;X(k)\geq \gamma \end{array}\right..$$

PO is therefore expressed in percentage as

(7) $$\mathrm{PO}=\left(\frac{L_{b}}{K}\right)\times 100,$$

where $L_{b}$ is the total number of data points at which $b_{k}=1$, such that

(8) $$L_{b}=\sum_{k=1}^{K}b_{k}.$$

**Figure 2:**
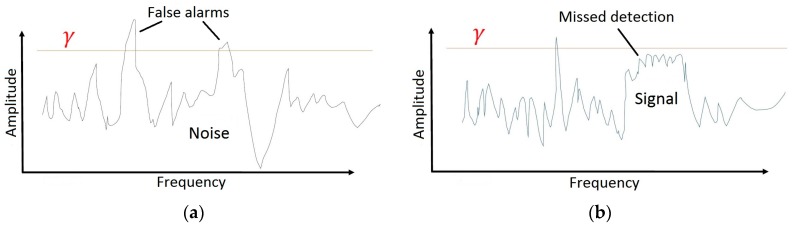
Effect of Percent Occupancy (PO) threshold setting: (**a**). If the threshold is set too low, the likelihood of false detection will increase; (**b**) If the threshold is set too high, the likelihood of missed detection will increase.

There are many competing techniques for the determination of γ. Uncertainty in the computation of PO is related to the threshold that defines the minimum power needed in a bin to achieve occupancy [20]. Figure 2 illustrates the importance of the γ value to a PO calculation. A poorly defined γ value will lead to an incorrect perception of the spectral opportunity. A common practice is to set a threshold on the basis of the noise floor of the system, assumed to be additive white Gaussian noise (AWGN). One consistency among the various methods is that the calculation of γ is typically calibrated based upon an estimate or a measurement of the system noise floor; however, system noise may vary over time so calibration data may not always be accurate or consistent. The validity of PO values hinges on the proper selection of the γ value that is used in Equation (6).

PO is a standard metric used to characterize the spectral capacity of the RF environment as it provides a general understanding of the openness or “clarity” of a channel [27]. For instance, if a channel has a high PO, it is likely that the opportunity within that channel is limited, while a low PO indicates more channel availability. While PO begins to provide an understanding of the spectral activity, further statistical analysis can reduce spectral uncertainty.

### 2.2. Total Signal and Noise Energy

Total signal energy (in joules) is defined as the the signal energy over the full period of time within a data collect, τ. It is computed as

(9) $$E_{x}=\int_{0}^{\tau }{\left|x(t)\right|}^{2}dt=\sum_{n=1}^{N}{\left|x(n)\right|}^{2}\Delta t,$$

wherein the impedance is assumed to be 1 Ω, by convention [28].

In a similar fashion, the total noise energy is given by

(10) $$E_{n}=\int_{0}^{\tau }{\left|x_{n}(t)\right|}^{2}dt=\sum_{n=1}^{N}{\left|x_{n}(n)\right|}^{2}\Delta t.$$

An essential BW is a finite band that contains the majority of the signal power [28]. Therefore, power outside of the essential BW can be considered negligible. Within an essential BW, we have

(11) $$E_{x}\approx \frac{1}{2\pi }\sum_{k=1}^{K}{\left|X(k)\right|}^{2}\Delta \omega ,$$

and

(12) $$E_{n}\approx \frac{1}{2\pi }\sum_{k=1}^{K}{\left|X_{n}(k)\right|}^{2}\Delta \omega .$$

In terms of dBJ (decibels relative to 1 joule), we can express, using Parseval’s theorem,

(13) $$E_{x,n}(\mathrm{dBJ})=10\log_{10}E_{x,n}.$$

A high total signal energy relative to the total energy of the spectral noise corresponds to one or more signals being present in that channel. While total signal energy and PO can be used in conjunction to portray the spectral capacity and density, an understanding of the distribution of the power and energy within the band is still lacking.

### 2.3. Power Spectral Entropy

The power spectral entropy (PSE) is most simply a measurement of the “uncertainty” of a power spectrum. PSE is maximized if the signal is AWGN and the value of entropy decreases as the distribution becomes less random, thus providing an indication of the characteristics of signals within a band. Thus, entropy is a measure of dispersion. PSE is robust to noise uncertainty and highlights minor deviations from AWGN in the spectrum, as entropy has a large dynamic range in the frequency domain [29].

PSE is calculated using an estimate of the probability density function (pdf) of the signal spectrum, $p_{\Psi }(X)$. The histogram method is used to estimate the pdf for PSE [30]. To attain an empirical estimate the pdf, we will first consider a PSD signal, X(k) denoted in Equation (3). The minimum and maximum values of X(k) are denoted as $X_{\min}$ and $X_{\max}$, respectively. This range of values is then divided into a discrete number of bins, Ψ, with a bin size $\Delta \psi =\left(X_{\max}-X_{\min}\right)/\Psi$, where Ψ is a value set based on the discrete number of output samples from the receiver.

Let $\kappa_{m}$ denote the number of X(k) values that correspond to the *m*-th power bin, i.e., those that lie in the range $\left[X_{\min}+(m-1)\Delta \psi ,X_{\min}+m\Delta \psi \right]$. Obviously, $K=\sum_{m=1}^{\Psi }\kappa_{m}$. The empirical pdf estimate using the histogram method is

(14) $$p_{\Psi_{m}}(X)=\frac{\kappa_{m}}{K}\;\mathrm{for}\;X_{\min}+(m-1)\Delta \psi <X(k)\leq X_{\min}+m\Delta \psi ,\;\forall m=1,2,\ldots ,\Psi .$$

An empirical estimate of entropy is therefore

(15) $$\begin{array}{ll} H(X) & =-\sum_{m=1}^{\Psi }\left(\frac{\kappa_{m}}{K}\right)\ln\left(\frac{\kappa_{m}}{K}\right)\\ & =-\sum_{m=1}^{\Psi }p_{\Psi_{m}}\ln p_{\Psi_{m}} \end{array}.$$

PSE is useful in determining portions of the spectrum with high and low activity. Consequently, PSE is a robust detector of white noise and can be used to reduce a radar’s spectral footprint. This concept is discussed more in Section 3.2. The metrics PO, total average power, and PSE all characterize the spectrum; however, none of these statistics provides an understanding as to a radar’s ability to detect a target in a given RF environment. SINR, discussed next, is directly related to a radar’s probability of detection.

### 2.4. Signal-to-Interference-Plus-Noise Ratio (SINR)

The SINR of a matched-filter response is the ratio of the signal energy to averaged interference plus noise power [31]. SINR is defined as

(16) $$\mathrm{SIN}R=\frac{E_{x}}{P_{n}+P_{I}},$$

where $P_{I}$ is the radio frequency interference (RFI) power.

Results have shown that the SINR is directly correlated with the probability of detection, $P_{D}$, and inversely corrected with both the probability of false alarm, $P_{F}$, and the probability of missed detection, $P_{MD}=1-P_{D}$.

### 2.5. Pulse Bandwidth

Radar range resolution is defined by a radar’s ability to distinguish targets that are separated in range from the radar. When performing matched filtering, range resolution is dependent on the width of the pulses produced by the cross-correlation function. The matched filter technique allows for a lengthened pulse width in the time domain (thus a large radiated energy), while maintaining a narrow range resolution. In radar, matched filtering occurs when cross correlation is performed between the transmit signal and received spectrum.

For frequency-modulated radars, the range resolution ΔR is inversely proportional to the pulse bandwidth (BW), as follows:

(17) $$\Delta R=\frac{c}{2B},$$

where B is the pulse BW and c is the speed of light [32]. Thus, for better resolution, BW should be as large as possible.

## 3. Multi-Objective Functions for Optimal Sub-Band (OSB) Selection

### 3.1. Spectrum Sensing Multi-Objective (SS-MO)

The spectrum sensing multi-objective (SS-MO) is a weighted sum multi-objective optimization algorithm (WSMO) [12]. The radar operation criteria driving the SS-MO are twofold: maximize SINR and maximize pulse BW (i.e., minimize range resolution). The fundamental premise of the SS-MO is that radar performance can be maximized based on the RF environment through the selection of an optimal sub-band (OSB) for transmission with both a high SINR and a high BW. Many cognitive radar schemes incorporate feedback from the RF environment to operate in frequency sub-bands of high SINR. The task of detecting sub-bands with both high SINR and a large BW, however, is not as trivial as it may seem, especially when taking into consideration the time constraints of the decision process.

In the case of SS-MO, noise plus interference energy is calculated based on the collected spectrum data in a real-time manner. An FFT is performed on the digitized I/Q spectrum data producing the frequency domain samples shown in Equation (3). The average power for each frequency bin k is defined as

(18) $$\theta_{k}=P_{n}(k)+P_{I}(k)={\left|X(k)\right|}^{2}.$$

**Figure 3:**
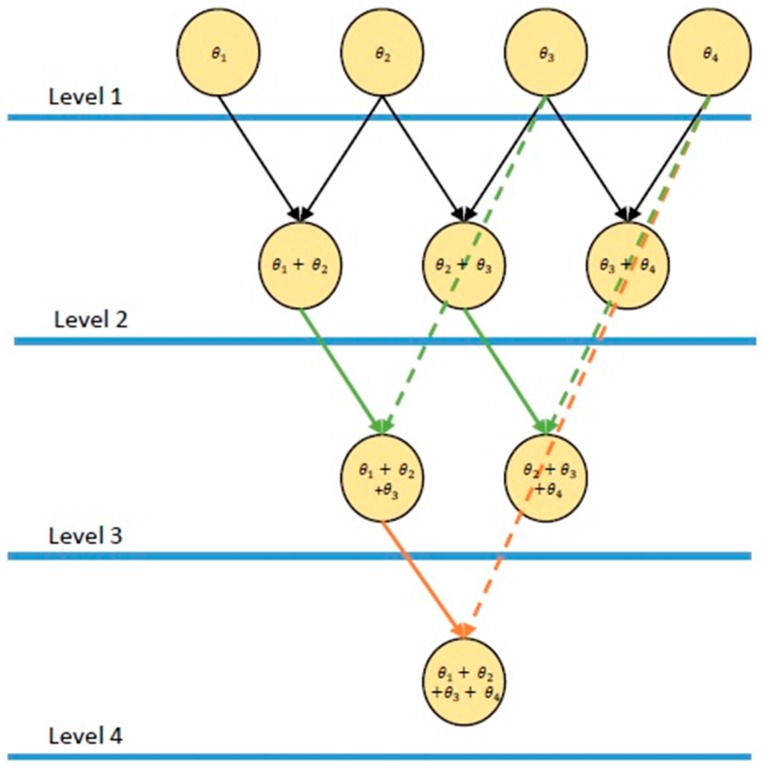
A four-level representation of the binomial summation technique used in spectrum sensing multi objective (SS-MO) and spectrum sensing multi-objective with brute force PSE (SS-MO-BFE) for the brute force calculation of noise plus interference power in the spectrum. Ultimately, the number of levels would correspond to the number of samples in the spectrum.

To compute brute force noise plus interference power efficiently, binomial summations are performed similarly to the computation of a Pascal triangle [33]. The power of each individual sample, $\theta_{k}$, is calculated in the first level of the triangle. In the next level, the power in each adjacent bin from the first level are added together. In the following levels, energy from level 1 is summed with power from the preceding level, as follows:

(19) $$P_{i,j}=\left\{ \begin{array}{ll} \theta_{j}, & i=1,\;j=1,\ldots ,K\\ P_{i-1,j}+P_{1,i+j-1}, & i=2,\;j=1,\ldots ,K-i+1\\ .\\ .\\ P_{i-1,j}+P_{1,i+j-1}, & i=K,\;j=1 \end{array}\right.,$$

where the level number is i and the element location for the *i*-th level is j [12]. Figure 3 is a representation of the summation method employed for a four-level data set.

The first objective function is equivalent to SINR, given by

(20) $$Z1_{i,j}=\frac{E_{s}}{P_{i,j}},$$

where $E_{s}$ is the signal power. The second objective function is the inverse of the range resolution since a lower value of ΔR equates to a higher likelihood of target distinction, from Equation (17). Therefore, the second objective function is defined as

(21) $$Z2_{i,j}=\frac{2\beta_{i,j}}{c},$$

where $\beta_{i,j}$ is the sub-band bandwidth. These values are normalized to their maximum values that are based on system considerations to yield dimensionless quantities $\hat{Z}1_{i,j}$ and $\hat{Z}2_{i,j}$, respectively.

A weighted combination of the two normalized objective functions yields

(22) $$Z_{i,j}=\alpha \hat{Z}1_{i,j}+(1-\alpha )\hat{Z}2_{i,j}.$$

where 0≤α≤1 is the weighting parameter. The weighting parameter α is adjusted to emphasize one objective function over the other and is dependent on the radar application. For example, it would be beneficial to set α≤0.5 for radar applications that require a high range resolution with large bandwidths. Since the goal of the proposed research is to investigate multi-objective optimization for arbitrary radar applications, we select α=0.5 in this develop to provide equal priority to each weighting function.

The maximum $Z_{i,j}$ provides the optimal (i,j) values given by

(23) $$(i^{*},j^{*})=\underset{i,j}{\mathrm{arg max}}Z_{i,j},$$

where the constraints are

(24) $$Z1_{\min}\leq \frac{E_{s}}{P_{i,j}},$$

(25) $$Z2_{\min}\leq \frac{2\beta_{i,j}}{c}.$$

**Figure 4:**
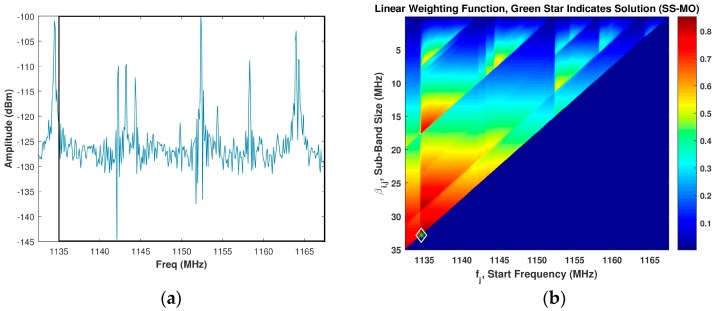
(**a**) Assumed spectrum; (**b**) Corresponding SS-MO linear weighting function, wherein the green star indicates the optimal sub-band OSB value. The black box in Figure 4a depicts the SS-MO solution.

In our analysis, the value used throughout for $Z1_{\min}$ was 0 dB and the value used throughout for $Z2_{\min}$ was 0.15 m which corresponds to a $\beta_{i,j}$ value of 5 MHz. For a typical spectrum shown in Figure 4a, the intensity image of the linear WSMO function, $Z_{i,j}$, for the SS-MO is shown in Figure 4b. The green star in Figure 4b indicates the pixel corresponding to the selected OSB for transmission, as determined by Equation (23), which in our case is a sub-band bandwidth of 35 MHz at a start frequency of 1135 MHz.

The SS-MO technique is designed with the intent of defining OSBs for a cognitive radar transmission. While the SS-MO moves towards optimizing a radar’s capabilities in a congested environment, it does not take into account the operational needs of the primary users. In Section 3.2, we outline a technique for detecting continuous sub-bands of the spectrum with limited activity. In Section 3.3, we develop a WSMO technique that includes a third PSE weighting function with the goal of reducing the RFI that is caused by the radar system on other systems while maximizing the radar’s performance.

### 3.2. Spectrum Sensing with Brute Force Entropy (SS-BFE)

Co-channel interference can be defined as RFI within the same band as the channel of operation of an RF system [34]. The utility of PSE in an objective function for the determination of an OSB for radar transmission is to reduce co-channel interference caused by the radar on the other systems in the immediate environment. Brute force PSE combined with image processing techniques are used to find continuous sub-bands of white noise in the spectrum in the SS-BFE algorithm. The goal of the SS-BFE technique presented is to limit the co-channel interference caused by a radar, which in turn improves its electromagnetic compatibility (EMC) characteristics. EMC is defined based on the following parameters: (1) interference caused on other systems, and (2) susceptibility to interference from other systems.

In scenarios where the reception of electromagnetic energy is not detrimental to the radar’s performance, an algorithm, such as SS-MO, does not necessarily select an OSB with an adequate EMC. The SS-MO it does not take into consideration the RFI that is caused by the radar. Normalized PSE can be very robust at finding bands with minimal RFI, or high spectral opportunity.

**Figure 5:**
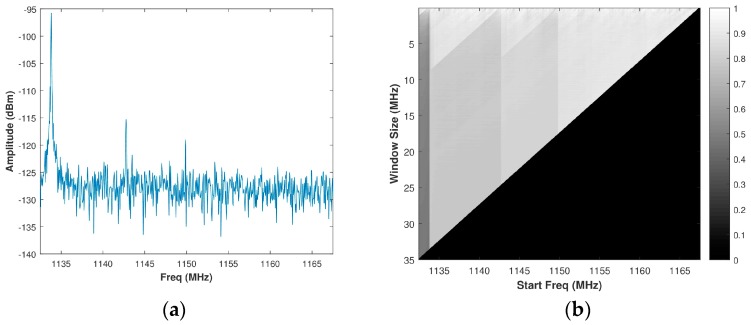
(**a**) Assumed signal spectrum; (**b**) Corresponding grey-scale normalized brute force power spectral entropy (PSE) image.

**Figure 6:**
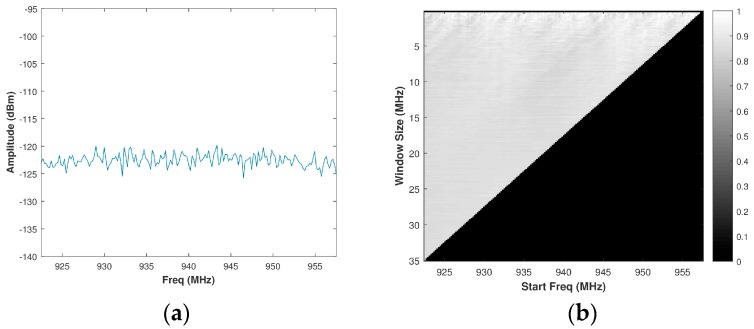
(**a**) Assumed noise spectrum; (**b**) Corresponding grey-scale normalized brute force PSE image.

Using Equation (15), a brute force calculation of PSE is performed for all possible window sizes, or sub-bands, of a spectrum to form a matrix H of size i×j, wherein i and j are defined as in Section 3.1. For the typical spectrum presented in Figure 5a, the resulting matrix of PSE intensities is then normalized to create a matrix or a grey-scale image is shown in Figure 5b. Since the normalized PSE value corresponds directly to the randomness of a sub-band of the spectrum, intuition would suggest selecting the value from the normalized brute force PSE matrix corresponding to the maximum PSE value. This approach, however, is not ideal. Consider a band of real system noise (collected from the SAS system outlined in Section 4), such as the one in Figure 6a. The histogram of the normalized PSE values was observed to be almost Gaussian distributed around a mean of 0.8626 with a very limited dynamic range when compared to that of a spectrum containing the signal(s). Its corresponding matrix of PSE intensities is depicted in Figure 6b. If one were to select an OSB purely based on the maximum PSE, a sub-band within a larger band of noise will be identified. The ideal approach is to select the maximum bandwidth sub-band of white noise in order to optimize radar operation (both SINR and range resolution) without causing additional co-channel interference to other systems. Selecting the full band of system noise rather than the sub-band of system noise would cause a Pareto improvement between the counter-parties of radar operation (defined by range resolution) and EMC (defined by PSE). Pareto efficiency is achieved when no counter-parties (range resolution and EMC) can change to cause an improvement among all of the parties [35]. Accordingly, Pareto improvement is a change that makes one of the counter-parties better off without hindering another counter-party [36].

Notice how the normalized brute force PSE image in Figure 5b is composed of several layers of isosceles right (45°-45°-90°) triangles. The bottom most point on each isosceles triangle corresponds to an index in which there is the maximum BW within a grouping of closely distributed PSE values. Consequently, the indices that correspond to the bottom most point on each isosceles triangle must be accurately determined. We present below a robust corner detection method for determining these points in a grey-scale image that was created from the brute force PSE matrix.

#### Application of Shi-Tomasi Corner Detector

Corner detection techniques are well developed in the field of computer vision and image processing due to their numerous applications, including object tracking and image correspondence. The utility of corner detection in image processing applications spans from the fact that corner points can be localized among images due to the stability of a corners features over varying viewpoints [37]. Localized areas in an image with high immediate changes in intensity in several directions can be defined as a corner.

Several approaches were examined for the determination of maximum BW within closely distributed PSE intensities on a brute force PSE grey-scale image. Two metrics considered when examining the utility of these corner detection techniques were: (1) corner detection ability, and (2) computational efficiency. Of the corner detectors considered, the Shi-Tomasi approach [38] was used. Although computationally intensive, it produced the most accurate results; thus, its superior performance outweighed its computational complexity of the algorithm. The Shi-Tomasi corner detection technique is derived from the Harris-Stephens corner detector [39]. The Harris-Stephens corner detection scheme is an efficient and effective method for detecting pixels of interest in an image, and it is based on the fact that there is a significant change in intensity in all directions surrounding a corner. Further mathematical details are available in [40].

**Figure 7:**
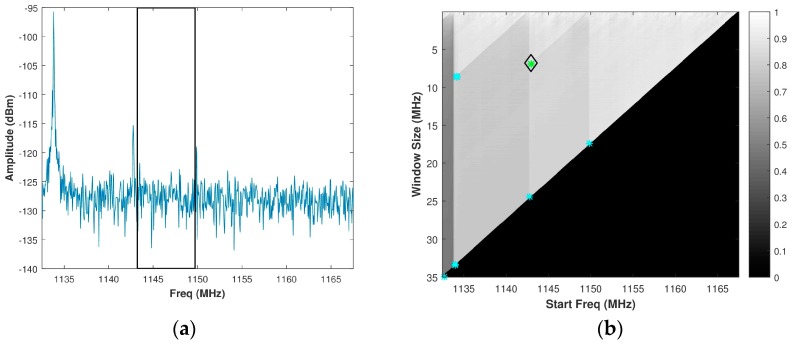
(**a**) Assumed signal spectrum; (**b**) Corresponding grey-scale normalized brute force PSE image showing corners detected by the Shi-Tomasi approach. The black box in Figure 7a depicts the spectrum sensing with brute force PSE (SS-BFE) solution.

Based upon the Shi-Tomasi approach, the corners detected are shown as colored stars in Figure 7b when applied to the normalized brute force PSE grey-scale image of Figure 5b. For reference purposes, the assumed signal spectrum from Figure 5a is reproduced in Figure 7a. Since the solution space does not allow for a range resolution of less than 0.15 m, corners for window sizes below 5 MHz are not considered. The green star is the optimal corner value determined, which results in a sub-band bandwidth of 6.5 MHz at a start frequency of 1143 MHz.

### 3.3. Spectrum Sensing Multi-Objective with Brute Force PSE (SS-MO-BFE)

As its name suggests, the SS-MO-BFE OSB identification technique is a WSMO function that considers the weighting functions for SINR, range resolution, and normalized PSE. Although the SS-BFE also considers all of these objectives, normalized PSE has a disproportionate amount of influence on the OSB selection. The driving concept of the SS-MO-BFE algorithm is that this technique provides a compromise between the two extreme solutions provided by the SS-MO and the SS-BFE. Since SS-MO-BFE is a WSMO, each parameter can be weighed individually thus allowing for additional flexibility. While the SS-MO defines an OSB with optimal radar performance and the SS-BFE defines an OSB in which a radar can perform, while causing minimal co-channel interference, the SS-MO-BFE is able to find a middle ground between both objectives.

The SS-MO-BFE takes the SINR and BW weighting functions used in the SS-MO algorithm, given by Equations (20) and (21), respectively, while adding a third normalized PSE objective for the determination of an OSB. The third objective function is

(26) $$Z3_{i,j}=H_{i,j}.$$

The WSMO equation used in the SS-MO-BFE technique is then

(27) $$Z_{i,j}=\alpha_{1}\hat{Z}1_{i,j}+\alpha_{2}\hat{Z}2_{i,j}+\alpha_{3}\hat{Z}3_{i,j},$$

where $0\leq \alpha_{i}\leq 1,\;i=1,2,3$ are the weighting parameters and $\sum_{i=1}^{3}\alpha_{i}=1$.

The values of $\alpha_{i}$ can be assigned on an application by applications basis. In our analysis, we assign equal weights to all of the objective functions considered, i.e., $\alpha_{1}=\alpha_{2}=\alpha_{3}=\frac{1}{3}$. Like the SS-MO, the OSB determined by the SS-MO-BFE is selected using Equation (23) with the redefined WSMO presented in Equation (27). The limiting factors for SINR and range resolution presented in Equations (24) and (25), respectively, apply to the optimal $Z_{i,j}$ indices in SS-MO-BFE as well.

**Figure 8:**
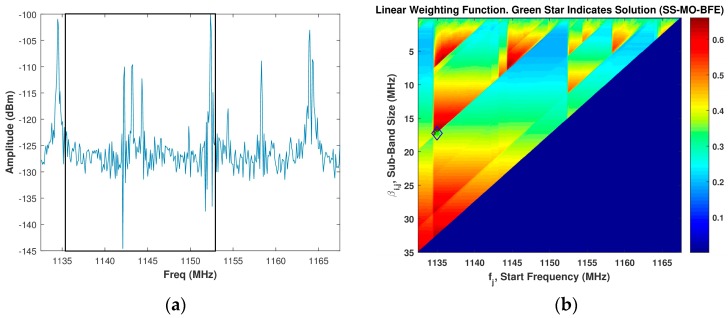
(**a**) Assumed spectrum; (**b**) Corresponding SS-MO-BFE linear weighting function, wherein the green star indicates the optimal sub-band (OSB) value. The black box in Figure 8a depicts the SS-MO-BFE solution.

Figure 8b displays an example image of the linear WSMO function, $Z_{i,j}$, for the SS-MO-BFE for a spectrum given in Figure 8a, which is the same spectrum used in Figure 4a. Due to the additional PSE weighting function considered, the SS-MO-BFE selected a smaller OSB than the SS-MO for this example spectrum. In this case, we obtain a sub-band bandwidth of 17.5 MHz at a start frequency of 1135.5 MHz.

## 4. Spectrum Analysis Solution (SAS) System Design Considerations

### 4.1. System Overview

The SAS is a high precision signal analyzer that is meant to perform energy detection for SS radar. There are several different techniques to sense the environment in a SS radar; however, none is quite as robust as energy detection. These detection theory methods include matched filtering, constant false alarm rates (CFAR), spectral correlation, cyclostationary detection, radio identification based sensing, and waveform based sensing [9].

**Figure 9:**
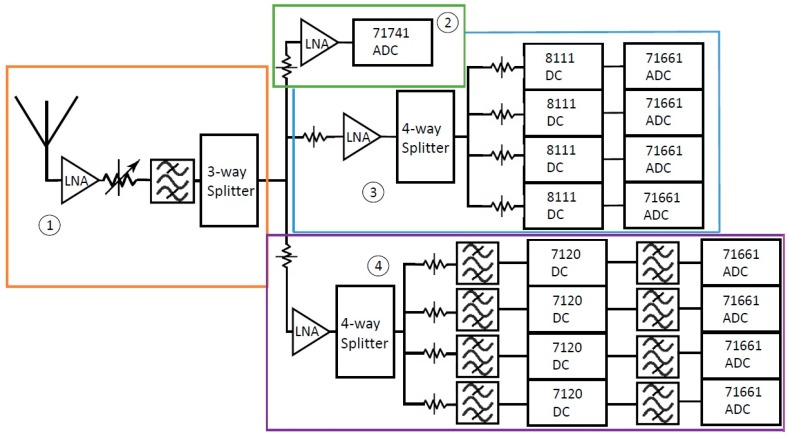
Block diagram of the Spectrum Analysis Solution (SAS) hardware. The block diagram is broken down into four subsections of the SAS which will be explained individually. The band pass filter blocks represent a series of filters.

As discussed in Section 1, the SAS is a nine-channel system that primarily operates in the ultra-wideband (UWB) range from ultrahigh frequency (UHF) to the S-band. Figure 9 shows a condensed block diagram of the SAS’s complete front-end leading to the digitization of the RF environment. The front end block diagram is broken down into four more subsections, which are individually boxed and numbered. The design goals of the SAS were to attain high resolution, high sampling rates, a large spurious free dynamic range (SFDR), high-speed processing capabilities, and a large storage capacity. The SAS consists of one wideband monitor channel and eight narrowband channels. The wideband monitoring channel is used to scan and characterize a broad region of the spectrum, ranging from 100 kHz to 1.8 GHz. The BWs of four of the eight narrowband channels are 50 MHz, while the BWs of the other four narrowband channels are 100 MHz. The BW of these channels is limited by the BW achievable by their downconverters. The 50-MHz BW applies to the channels containing the 8111 downconverters, while the 100-MHz BW applies to the channels containing the 7120 downconverters.

The wideband monitoring channel points the eight narrowband channels to areas of interest within the spectrum so that the narrowband channels can provide a higher resolution image of the spectrum. Areas of interest within the spectrum can be defined in many ways. In the case of the SAS, these are defined as those with high energy or activity. The benefit of the SAS is its ability to provide a high-resolution understanding of the spectrum without the trade-off of BW.

### 4.2. Hardware Implementation and Validation

Digital electronics play a key role in a radar’s ability to handle complex environments. Additional dynamic range is achieved by moving these digital components as close to the receiver as possible [41]. The core components of the SAS system consist of the Pentek downconverters and analog-to-digital converters (ADCs). A hardware validation was performed in order to better understand the SAS’s true capabilities beyond the vendor supplied specifications. A characterization of these core components was the first step to ensure a calibrated setup. Based on these characterizations, a front-end was designed to condition the signal. Extensive hardware testing of each component in the system was performed to ensure its proper performance.

#### 4.2.1. ADC and Downconverter Performance Validation

The ADCs and downconverters (DCs) are referenced by their Pentek model numbers. The 7120s and 8111 opt. 1-4 are downconverters, while the 71661 and the 71741 are narrowband and wideband ADCs, respectively. Table 1 outlines the general specifications of these DCs and ADCs.

In order to validate the ADCs and DCs, the frequency responses of the cards are considered. Automated tone sweeps generated by a Keysight N5171B EXG signal generator were input at the receive channel of each ADC individually in order to understand how well the digitized output of the ADCs match a controlled input. The SAS has been designed such that the narrowband channels (corresponding to the 71661 ADCs) observe the energy downconverted to the band of 200 to 250 MHz that has been aliased into baseband to avoid intermodulation (IM) products surrounding zero frequency. Since a large amount of aliasing was evident in each ADC card, a significant amount of energy from a tone outside of each ADCs’ frequency band was detected within that band. This resulted in the need for anti-aliasing filters prior to the ADCs.

The ADCs act as the bottle neck for the frequency resolution of the system as the other components are analog rather than digitized. The Pentek 71741 wideband ADC has a sampling rate of 3.6 GSa/s, while the narrowband 71661 ADCs have a sampling rate of 200 MSa/s. To reiterate, the purpose of the narrowband channels is to display a high-resolution image of the spectrum where a wideband channel cannot. To gain a better perspective on the overall performance of the system, an estimate of the narrowband resolution was attained through “two-tone testing”. One tone remained static, while the other tone was swept past the static tone with a step size of 1 kHz. The concept behind the test is that the ADC has a minimum resolution of the difference between the tone frequencies the last time the two tones could be distinguished. Note that the number of points collected by the ADC (which is variable for the SAS) affects the frequency resolution and noise floor of the SAS. The testing was performed with the standard 2^16^ points for the 71661 narrowband ADCs. The number of points per spectra is variable, however 2^16^ is the standard value used for the narrowband channels. The experimental resolution of the 71661 ADC was approximately 3 kHz, which is high resolution by today’s standards for the proposed application.

The downconverter operation was validated by looking at the frequency response of the downconverters with variable settings. The RF input of each downconverters was connected to a signal generator, while the IF output was connected to a spectrum analyzer. A single tone was sent into the downconverters while the tuning bands and attenuation were changed. The amplitude and frequency of the tone were swept beyond the tuning band to identify the ability of the internal filtering. The internal filtering for the 8111 downconverters were very tight and performed well over a broadband view; however, a need for further anti-aliasing filtering was identified for the 7120 downconverter channels.

#### 4.2.2. Front End Design

Several iterations of front end design were performed before reaching the current state. Co-site interference is defined as interference on a channel that is created by RF energy in the environment that is outside of that channel’s operational BW [34]. The SAS’s front-end mitigates co-site interference with minimal degradation of each channel’s bands of interest. The National Telecommunications and Information Administration (NTIA) produced studies of the interference that out-of-band (OOB) emissions can cause in Next-Generation Weather Radars (NEXRADs) [42]. With this study in mind, the SAS’s front-end was designed to reduce co-site interference by reducing OOB interference. All of the components in the SAS are commercial off-the-shelf (COTS) and are connected using low-loss coaxial cables. The remainder of Section 4.2.2 describes the design of the four hardware subsections labeled in Figure 9 individually in greater depth.

##### Front End Block 1

**Figure 10:**
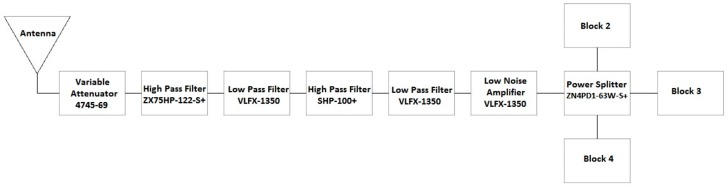
Detailed block diagram of Block 1 in Figure 9.

A detailed block diagram of Block 1 of the SAS is shown in Figure 10. Part numbers correspond to those manufactured by Mini-Circuits (https://www.minicircuits.com), unless otherwise specified. Block 1 is composed of an antenna, a series of filters, an amplifier, and a power splitter. The primary function of Block 1 is to capture and filter the RF energy across the full scope of the SAS. The antenna at the front end should have a BW that supports the full BW of the SAS. The Schwarzbeck BBHA 9120 E double ridged broadband horn antenna (http://schwarzbeck.de/Datenblatt/k9120e.pdf) was used in our SAS design. The antenna acts as an additional band pass filter (BPF) at the front end. Filters ZX75HP-122-S+, VLFX-1350, and SHP-100+ combine in series to act as a BPF that filters across the band of 100 kHz to 2 GHz. Additional amplification is added with the low noise amplifier (LNA), ZKL-2R7+. Finally, the signal power is split between the three adjoining blocks of the SAS using a power splitter. All of the power splitters used in the SAS are the ZN4PD1-63W-S+.

**Figure 11:**
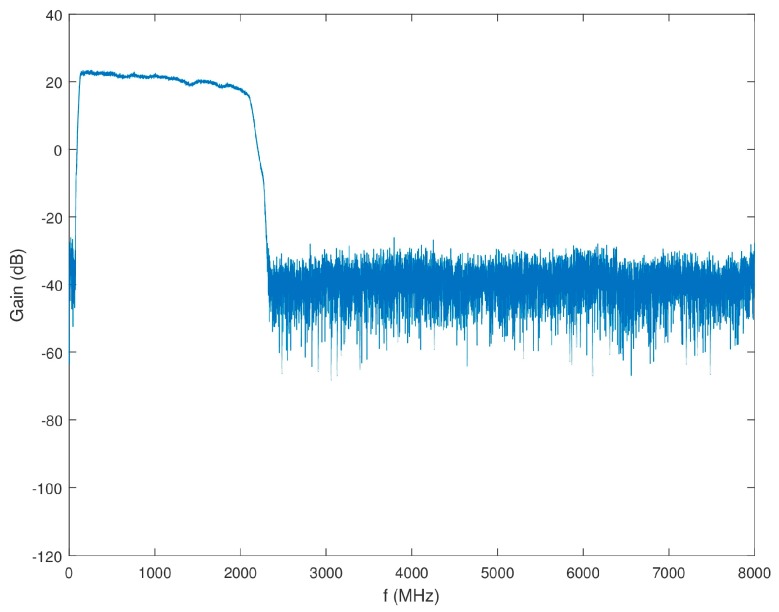
Gain of Block 1 (minus antenna) as a function of frequency.

The overall gain of Block 1 excluding the antenna, but including the power splitter is shown in Figure 11. It was also confirmed that the isolation between the power splitter output channels was higher than 20 dB across the desired band.

##### Front End Block 2

**Figure 12:**
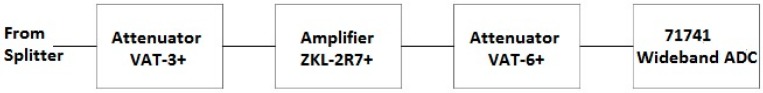
Detailed block diagram of Block 2 in Figure 9.

**Figure 13:**
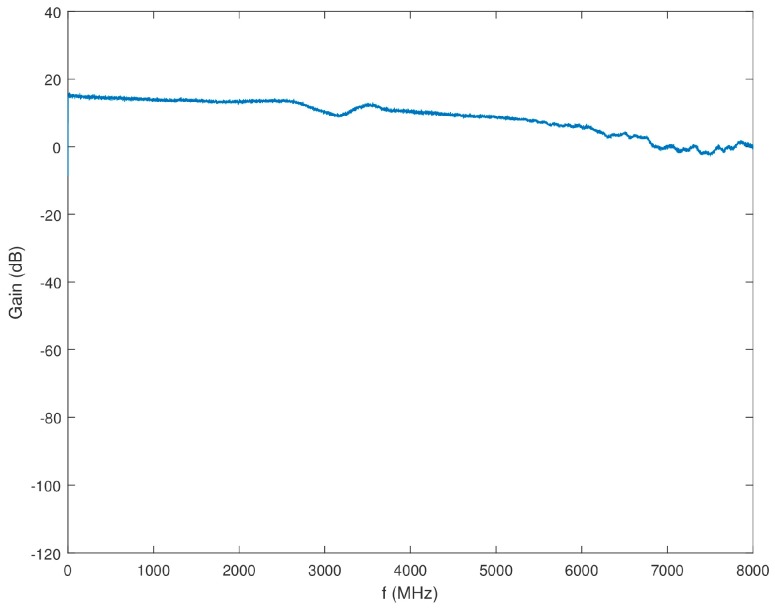
Gain of Block 2 as a function of frequency.

Block 2 of the SAS represents the 1.8-GHz wideband channel. A comprehensive block diagram of Block 2 is shown in Figure 12. As the input to Block 1 filters the signal across the full BW of the SAS, no additional filtering is required prior to the 71741 wideband ADC. A 3-dB attenuator, VAT-3+, is placed at the output of the splitter to increase the splitter isolation. A ZKL-2R7+ LNA amplifies the signal to overcome some of the power loss in the splitter and attenuator. A 6-dB attenuator, VAT-6+, is placed prior to the 71741 wideband ADC to again increase channel isolation. Figure 13 shows the gain of the series of components that compose Block 2.

##### Front End Block 3

**Figure 14:**
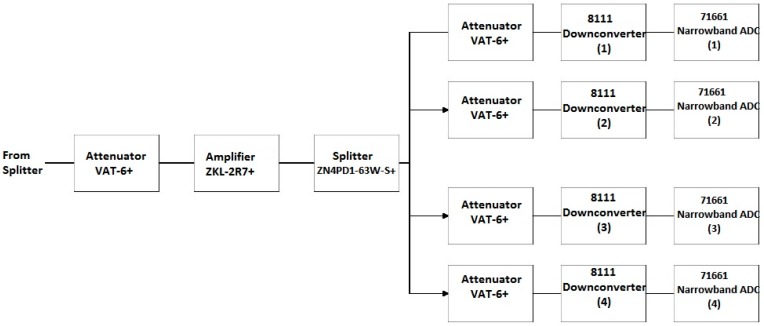
Detailed block diagram of Block 3 in Figure 9.

**Figure 15:**
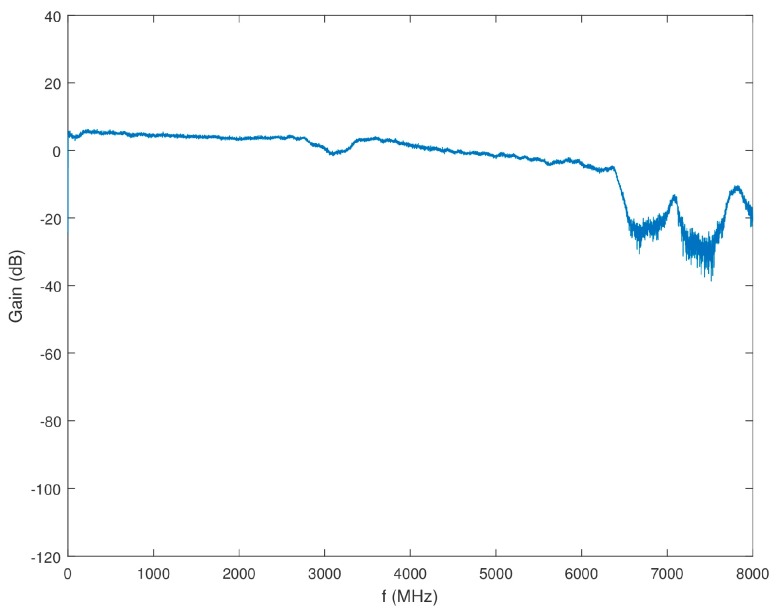
Gain of Block 3 as a function of frequency.

Block 3 of the SAS includes four of the eight narrowband channels corresponding to the 8111 opt. 1–4 downconverters. A comprehensive block diagram of Block 3 is shown in Figure 14. The downconverter testing, as discussed in Section 4.2.1, led to the conclusion that the internal filter bank of the 8111 opt. 1-4 downconverters was adequate for the performance of these channels. Therefore, no additional anti-aliasing filtering is applied to these channels in Block 3. A 6-dB, VAT-6+, attenuator follows the splitter from Block 1 to increase the splitter isolation. Next, the ZKL-2R7+ LNA amplifies the signal to cancel some of the loss in the splitter and attenuators. The signal is again split between the four narrowband channels with added attenuation to increase channel isolation. The gain of the series of components that compose Block 3 leading to the inputs of the 8111 opt. 1-4 downconverters is displayed in Figure 15.

##### Front End Block 4

**Figure 16:**
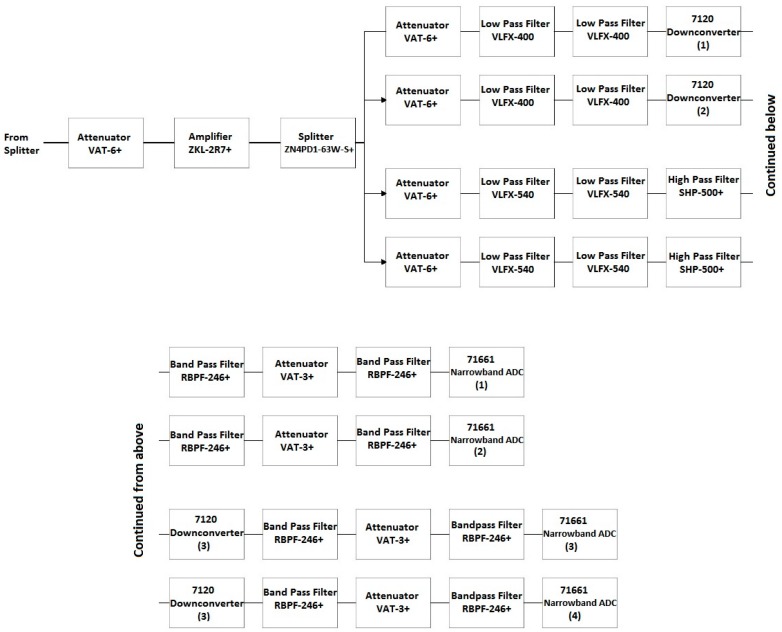
Detailed block diagram of Block 4 in Figure 9.

Block 4 of the SAS includes four of the eight narrowband channels corresponding to the 7120 downconverters. A comprehensive block diagram of Block 4 is shown in Figure 16. In Section 4.2.1, the need for additional anti-aliasing filtering surrounding the 7120 downconverters was established in order to prevent unwanted co-site interference. While the 7120 downconverters have a total BW of 35 to 4400 MHz, developing a filter bank to support this BW is unnecessary as the channels containing the 8111 opt. 1-4 are capable of providing a high-resolution image of the spectrum from 800 to 2100 MHz. Therefore, a narrower filter scheme was implemented for the narrowband channels contained in Block 4 focused on the lower frequency range of the SAS. The first two channels containing the 7120 downconverters have been re-purposed for use in the band of 200–550 MHz and the second two channels containing the 7120 downconverters have been re-purposed for use in the band of 500–800 MHz.

**Figure 17:**
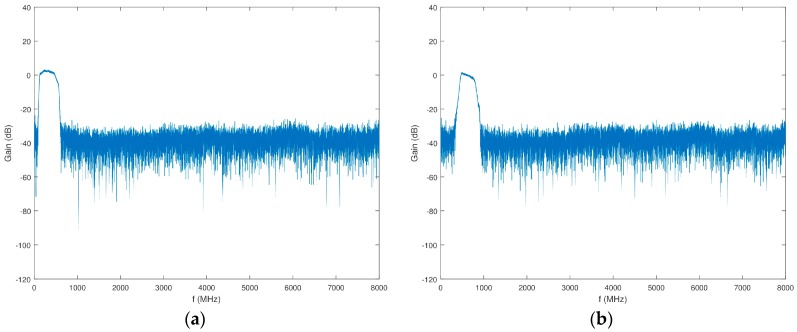
Gain of Block 4 prior to the 7120 downconverters as a function of frequency. (**a**) Channels 1 and 2; (**b**) Channels 3 and 4.

Similar to the scheme in Block 3, the input of Block 4 features an attenuator (VAT-6+), an LNA (ZKL-2R7+), a splitter (ZN4PD1-63W-S+), and another attenuator (VAT-6+). The first two channels have two low pass filters (LPFs) (VLFX-400+) prior to the 7120 downconverters. The second two channels have two LPFs in series with a HPF (VLFX-540 and SHP-500+, respectively) prior to the 7120 downconverters. Additional anti-aliasing filtering and isolation in the form of two filters and an attenuator (RBPF-246+ and VAT-3+, respectively) are placed between all four 7120 downconverters and 71661 narrowband ADCs. Figure 17 shows the gain of this series of components in Block 4.

### 4.3. Software Implementation

**Figure 18:**
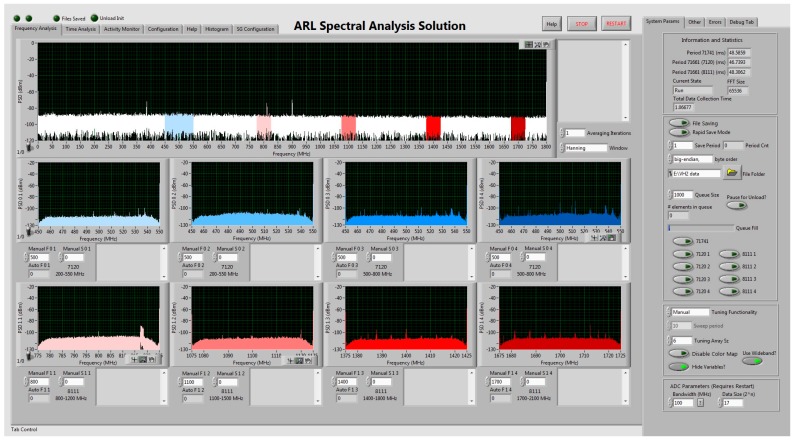
The SAS LabVIEW GUI front panel.

The basic implementation goal of the SAS’s software was to stream data as rapidly as possible. The duty cycle of collection time to collection time plus processing time was considered when designing the SAS’s software. This duty cycle $D_{cp}$ is denoted as

(28) $$D_{cp}=\frac{\tau_{c}}{\tau_{c}+\tau_{p}},$$

where $\tau_{c}$ is the time spent collecting and $\tau_{p}$ is the time spent processing in a real-time system. In order to maximize this duty cycle, the SAS performs minimal active processing. The primary purpose of the current version of the system is to provide data for post-processing. A ReadyFlow C program primarily written by Pentek controls the ADCs and downconverters. A LabVIEW graphical user interface (GUI) calls the modified ReadyFlow C programs provided by Pentek in order to initialize and write certain card parameters. Some of these card parameters include: number of samples, sensitivity (variable attenuation), and tuning frequency. The ADCs produce I/Q data through a Hilbert transform of the time domain signal. A Hilbert transform produces the orthogonal signal to the receive signal. This operation is performed on the field programmable gate arrays (FPGA) that are located on the ADC’s printed circuit board (PCB). The I/Q data are therefore streamed directly to the SAS computer via a PCI-express 8X cable. The GUI then opens up a TCP connection with the cards and begins the process of streaming 16 bit I/Q data from the individual channels. The GUI allows for some real-time processing along with actively updated frequency and time domain graphs for each channel. The SAS GUI, shown in Figure 18, has the capability to perform the following real-time functions:

1. Perform a fast Fourier transform (FFT) on the I/Q data
2. Display time and frequency domain spectra to the front panel for all nine channels
3. Auto-tune, sweep or manually adjust the instantaneous bands of each channel individually
4. Manually adjust the attenuation and number of samples of each channel individually
5. Monitor the relative activity of the spectrum for the wideband channel
6. Display an active binary histogram of the spectral data for all nine channels

Since the real-time processing capabilities of the SAS are limited, the SAS provides an option to save data to a binary file that allows for post-processing. Parallel processing techniques were implemented to streamline the processing and maximize the GUI’s efficiency. A rapid save mode was also implemented in which the SAS GUI forgoes all active processing to maximize $D_{cp}$. When the SAS is in rapid save mode, I/Q data are streamed directly from any combination of the nine channels (selected by the user) and written to binary files. Along with the exclusion of active processing, no datum is displayed to the screen when the SAS is in rapid save mode.

## 5. Data Collection, Generation, and Analysis

Data were collected using the SAS in several instances in order to build a database of spectra to perform statistical spectrum analysis. To gain perspective on the spectrum, an ambient data collection was performed. Next, closed loop testing was performed in which an arbitrary waveform generator (AWG) fed spectra directly into the SAS via cable. Closed loop testing was performed using a pseudo-random generation technique, which relied on a combination of quantitative and qualitative observations of the ambient spectrum data.

We also present statistical analysis of the pseudo-random data collected by the SAS utilizing the multi-objective functions for OSB selection defined in Section 3 that are based on the statistics discussed in Section 2. It should be noted that the pseudo-random WF data discussed herein have been decimated to decrease the necessary processing time. A Hamming LPF has also been applied to these spectra to reduce high frequency noise. The established SS-MO algorithm from Ref. [12] is used as a base line for comparison with the performance of the new algorithms developed in this paper.

### 5.1. Ambient Data Collection

Ambient SAS data were collected in a suburban area with a medium to high density of people. Rather than utilizing the SAS’s auto-tune or sweep functionality for this data collection, the narrowband, high resolution channels were focused on bands in the spectrum in order to simplify the analysis. A more continuous time picture of the spectrum is achieved with a static instantaneous band as well.

**Figure 19:**
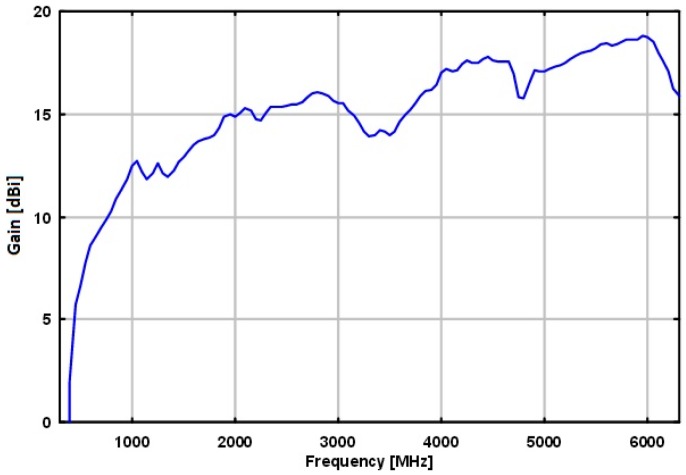
Gain versus frequency of the Schwarzbeck BBHA 9120 E double ridged broadband horn antenna.

The Schwarzbeck BBHA 9120 E double ridged broadband horn antenna was used and was selected because of its broadband and high directivity performance. This antenna performs adequately in the lower frequency bands without sacrificing performance among the higher frequencies. Figure 19 shows the gain of the antenna relative to an isotropic radiator.

**Figure 20:**
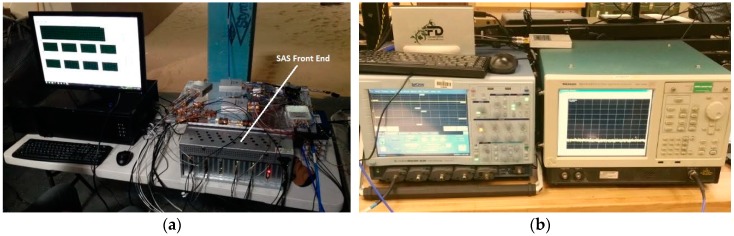
(**a**) SAS system. (**b**) Oscilloscope and real-time spectrum analyzer (RSA).

The input signal was split directly into the SAS’s front-end as well as into a LeCroy WaveMaster 8620A oscilloscope and a Tektronix RSA6114A real-time spectrum analyzer (RSA) for data verification purposes. Figure 20 shows photographs of the ambient data collection setup. The data collected is used as motivation for more controlled algorithm testing.

### 5.2. Statistical Analysis of the Ambient Data

A statistical analysis of the spectra from the ambient data collection was performed to guide the generation of spectra for closed loop testing. Two statistical metrics were considered: PO and total signal energy (or alternately, total average power $P_{s}$) and PO, as derived in Section 2.1 and Section 2.2, respectively.

In Section 2.1, $b_{k}$ is calculated on the basis of a threshold, γ. It should be noted that the threshold γ is used to characterize the collected data in terms of total average power versus percent occupancy. This characterization provides an assessment of the spectra processed by the multi-objective optimization algorithms (discussed below). For example, the algorithms would process high-power, narrowband interference different from low-power, wideband interference. The functionality of the multi-objective optimization algorithms themselves do not use thresholding since the algorithms search a solution space for the result.

Ambiguity in the determination of PO stems from the method of selection of γ. Several active noise floor estimation techniques can be used in defining γ. For the purpose of characterizing these spectra, the luxury of real-time noise floor computation was not afforded. Rather, the SAS was taken off-line and calibration data were collected. A 50-Ω termination was connected to the input of the front end of the SAS and a relatively long-term run sequence was executed. A measurement of the system’s noise floor over time was effectively taken. A point-by-point average of the calibration data in the linear frequency domain was then calculated at each tuning frequency in order to provide an estimate of the noise floor mean. The point-by-point average is calculated using

(29) $$\mu (k)=\frac{1}{N}\sum_{n=1}^{N}YY(k_{n},c),$$

where N is the total number of collections at each tuning frequency, YY is the matrix of linear frequency domain data that is defined by the frequency bin, k, and the collection number, n.

The PO threshold, γ, was then specified as

(30) γ=μ(k)+3σ,

where σ is the standard deviation of the point-by-point average. Setting γ to three standard deviations above the running average is appropriate because only 0.1% of AWGN will exceed γ.

**Figure 21:**
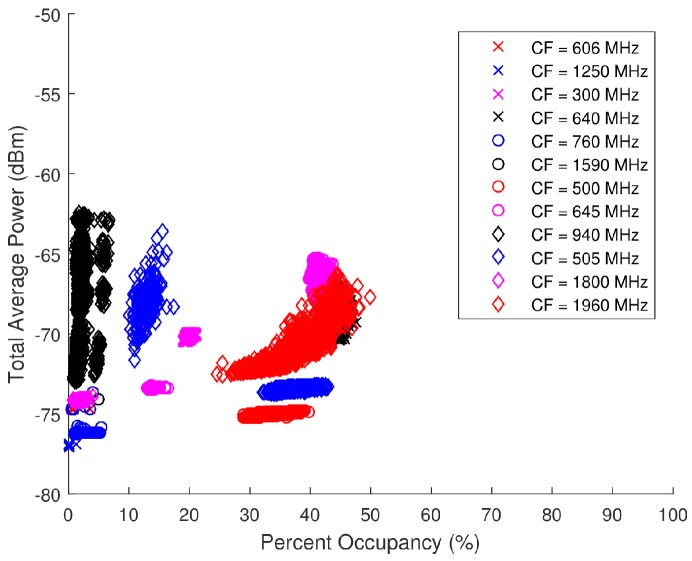
Scatter plot of the total average power versus percent occupancy of the spectra from the ambient data collection.

Figure 21 is a scatter plot of the total average power versus PO for the ambient data collected. This scatter plot breaks the points up into select sub-bands showing the individual distributions for specific center frequencies (CF). These sub-band distributions can therefore be cataloged based on FCC mandated applications. For instance, in Figure 21, there is a grouping of points around $\left(P_{s},\mathrm{PO}\right)$ = (−67 dBm, 45%) from a 50-MHz BW with a 1960-MHz CF. The corresponding allocated applications for a band of 1930 to 2165 MHz pertain to personal communication systems (PCS), 2-GHz mobile satellite services (MSS) uplink, space, fixed, and mobile. A combination of relatively high total average power and PO would support the assumption that these spectra are likely to contain one or more wideband signals. Due to the suburban location of the data collection, it can be surmised that cellular phones are the primary sources of the energy detected in this spectra, particularly Long-Term Evolution (LTE) mobile communication. A similar process can be used with the other sub-band distributions to speculate on the applications detected in the spectra.

Total average power and PO are not directly proportional; however the scattering is not completely random either. In many scenarios, a higher total average power will lead to a higher PO, although that is not always the case.

### 5.3. Closed Loop Testing

A pseudo-random database of spectra was compiled using the Agilent M8190A 12GSa/s Arbitrary Waveform Generator (AWG). The motivation for using a pseudo-random database of spectra is to attain an understanding as to the performance of the algorithms. A controlled spectra at the input of the SAS limits the complexity of the problem space, allowing for a more conclusive analysis as to the performance of the algorithms discussed. The signals produced by the AWG consisted of a pseudo-random combination of chirps (wide band) and sinusoids (narrow band).

**Figure 22:**
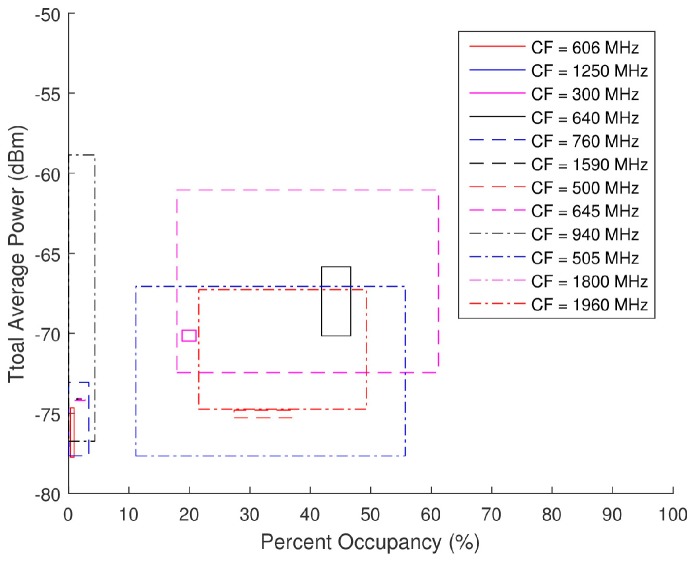
Range of total average power and PO calculated for each center frequencies (CF) of the ambient data collection from Equations (31) and (32).

During the closed loop testing, the spectra generated by the AWG were split into the SAS, LeCroy’s WaveMaster 8620A oscilloscope, and Tektronix RSA6114A real-time spectrum analyzer (RTSA). Only the data collected by the SAS are analyzed herein as the other systems were used for verification or separate testing purposes. Total average power and PO serve as the two input parameters that provide a basis for the generation of the pseudo-random spectra. These scatter plots presented in Figure 21 serve as the foundation on which the pseudo-random data sets were modeled. An analysis as to the distribution of the total average power and PO of each band (denoted by its specific CF) was performed to select spectra with similar distributions from the pseudo-randomly generated database. The range of acceptable total average power and PO for each ambient spectra is displayed in Figure 22 and Table 2 along with the corresponding waveforms (WF) selected from the pseudo-random database. 

**Figure 23:**
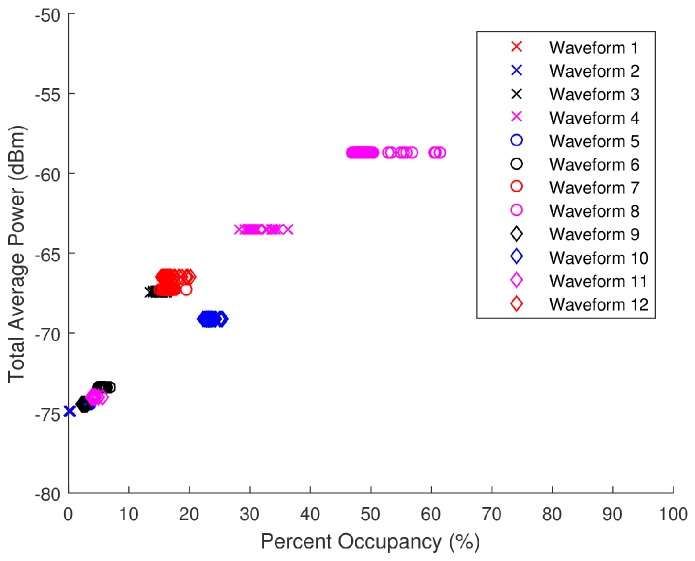
Scatter plot of the total average power versus percent occupancy of the pseudo-randomly generated spectra data collected by the SAS.

These total average power and PO ranges were calculated as

(31) $$[P_{s,\mathrm{upper}},P_{s,\mathrm{lower}}]=\mu_{P_{s}}\pm 3\sigma_{P_{s}},$$

and

(32) $$[{\mathrm{PO}}_{\mathrm{upper}},{\mathrm{PO}}_{\mathrm{lower}}]=\mu_{\mathrm{PO}}\pm 3\sigma_{\mathrm{PO}},$$

respectively. Due to the extensive collection time and static nature of certain ambient spectra, the calculated ranges of total average power and PO had very small standard deviations making it difficult to find pseudo-randomly generated spectra that fell within the allotted ranges. In these circumstances, spectra with total average power and PO contiguous to the specified ranges were selected. Figure 23 shows the total average power versus PO scatter plot for the pseudo-randomly generated waveforms. There is considerably less jitter in Figure 23 as compared to that in Figure 21 as the pseudo-random WFs are not time-varying.

### 5.4. Comparative Analysis Utilizing the Pseudo-Random Database

The performance of the SS-MO, SS-BFE, and SS-MO-BFE algorithms are compared on the basis of the following: (1) Statistical performance, and (2) Computational efficiency. Since the algorithms’ computational efficiency has not been specifically optimized, the results from a computational standpoint are less significant. The data set used for the comparison are the twelve pseudo-randomly generated WFs outlined in Table 2. As outlined in Section 5.3, these WFs were fed directly into the input of the SAS. For the purpose of this section, fifty spectrum data per WF were analyzed.

### 5.5. Statistical Performance

**Figure 24:**
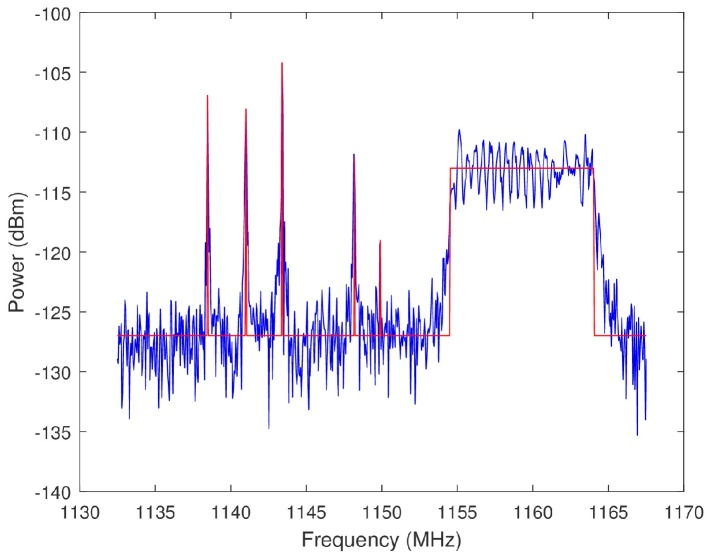
Plotted in blue is an example spectra from the closed loop data. Any frequency bin with an amplitude above −127 dBm on the red line corresponds to an emitter.

The performance metrics utilized in this analysis are SINR, BW (as it affects radar range resolution), and percent interference (PI). SINR and BW are defined in Section 2.4 and Section 2.5, respectively. PI is defined as the ratio

(33) $$\mathrm{PI}=\left(\frac{Em_{B}}{Em_{T}}\right)\times 100,$$

where $Em_{B}$ is the number of emitters within the OSB selected by an algorithm and $Em_{T}$ is the number of emitters in the total view of the wideband spectrum. Because the spectra were generated, all of the signals within the spectra are known. Therefore, an emitter is defined as any individual sinusoid or chirp within the band of a spectrum. The determination as to whether an emitter is within a select OSB is binary. The FCC Spectrum Policy Task Force defines harmful interference as “Interference which endangers the functioning of a radionavigation service or of other safety services or seriously degrades, obstructs, or repeatedly interrupts a radiocommunication service operating in accordance with these [International] Radio Regulations” [2]. The reasonable assumption is made that a radar transmission in an OSB overlapping less than 1% of a wideband signal does not degrade the primary user’s ability to perform its intended function. Therefore, any overlap of less than 1% is not considered interference. See Figure 24 for an intuition regarding the bounds of a signal classified as an emitter. The noise floor of the spectrum in Figure 24 is approximately −127 dBm. Any frequency bin with an amplitude above −127 dBm on the red line is declared an emitter. The PI quantifies the amount of co-channel interference that is caused by a radar transmission across the select OSB. An intuition for the spectral footprint, and to an extent the EMC, of a radar can be derived from PI.

An algorithm’s performance for each statistic is quantified through a root-mean-square error (RMSE) defined as

(34) $$\mathrm{RMSE}=\sqrt{\frac{1}{l}\sum_{i=1}^{l}{\left|e_{i}\right|}^{2}},$$

where

(35) $$e_{i}=\frac{O_{i}-D_{i}}{w_{i}}.$$

In Equation (35), $O_{i}$ is the metric’s best value, $D_{i}$ is the metric’s value as selected by the algorithm, and $w_{i}$ normalizes the error based on the total range of possible values. The best values ($O_{i}$) for PI, BW, and SINR are 0%, 35 MHz, and $\max(Z1_{i,j})$, respectively. A RMSE = 0 denotes a metric that has been optimized. In the case of BW, $O_{i}=35\;\mathrm{MHz}$ and $w_{i}=30\;\mathrm{MHz}$. For PI, $O_{i}=0.00\%$ and $w_{i}=100\%$.

**Figure 25:**
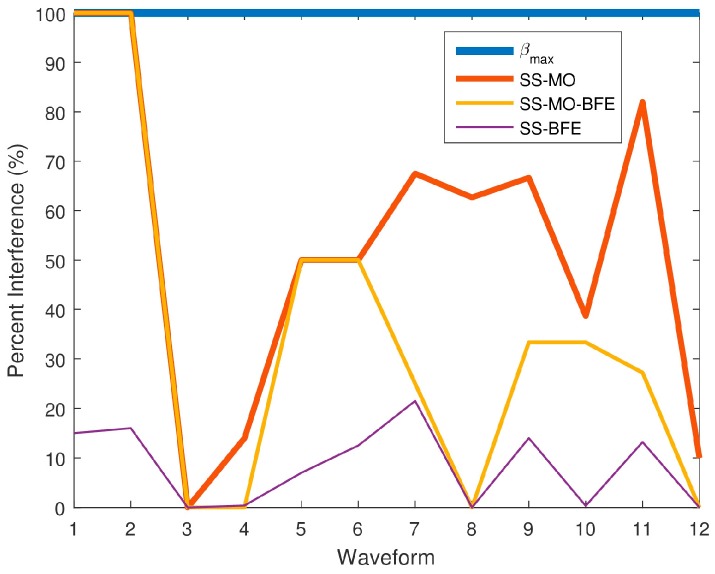
Average percent interference (PI) values for each SS technique for each pseudo-random waveform.

Figure 25 shows the PI results for all of the algorithms for comparison with the PI for $\beta_{\max}$, the maximum available BW yielding the best resolution. In Figure 25, the 50 spectrum datum collected by the SAS were averaged for each WF. Table 3 outlines the breakdown of emitters in each spectrum along with the average PI that is caused by the select OSB for each algorithm individually. Only once did all three algorithms (Waveform 3) have the optimal average PI of 0%, simultaneously resulting in an RMSE of 0. Of the three algorithms, the SS-BFE minimized PI most efficiently since its overall RMSE was the least, followed by SS-MO-BFE, and then the SS-MO whose RMSE was the highest. All three algorithms show a significant improvement over the PI of $\beta_{\max}$, which has a constant PI of 100%.

In the spectra corresponding to Waveform 4, the RMSE of SS-BFE was observed to be slightly greater than that of the SS-MO-BFE. However, the difference is minute, indicating a slight, insignificant variation in performance. It is worth noting that both spectra contain a chirp emitter. In OSBs containing wideband signals such as chirps, the PSE value can be inflated as the energy within the wideband signal can be almost uniformly distributed leading to poor OSB identification. This is not typically the case as the energy in most wideband signals are less uniformly distributed than a sub-band containing purely noise. This effect is amplified in a controlled input scenario as signal fading is not a factor. The SINR weighting function in SS-MO-BFE counteracts the high PSE values as wideband signals are typically high powered RFI.

**Figure 26:**
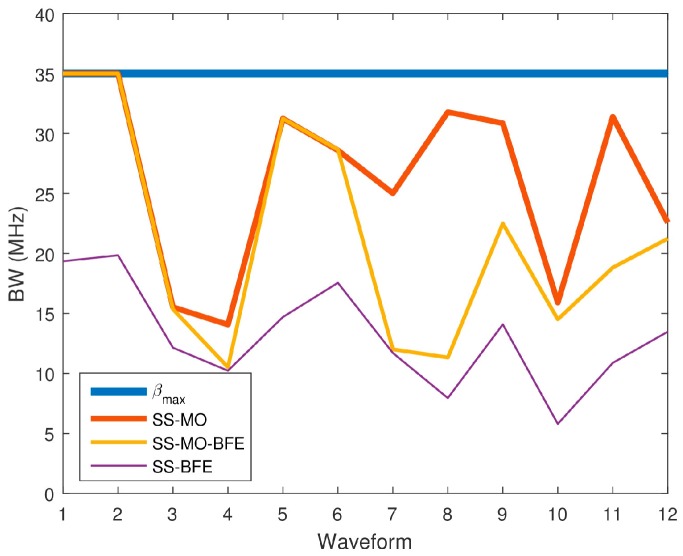
Average bandwidth (BW) values for each SS technique for each pseudo-random waveform.

Figure 26 compares each algorithm’s $\beta_{i,j}$ results averaged over each spectrum. Because range resolution is inversely related to BW, a high BW is desirable in order to achieve the best resolution. Table 4 shows the average $\beta_{i,j}$ value selected by each technique.

As anticipated, the SS-MO provides the best range resolution among all of the algorithms followed by the SS-MO-BFE, and then followed by SS-BFE over the complete dataset.

Of all three algorithms, the SS-MO provides the most weight for maximizing bandwidth as the weighting factor on the range resolution objective function, $Z2_{i,j}$, is α=0.5. This weighting factor is larger than that of the SS-MO-BFE, which uses a weighting factor of $\upsilon =\frac{1}{3}=0.33$ for $Z2_{i,j}$. Because SS-BFE is not a traditional WSMO function, the weight applied to the range resolution objective cannot be directly compared to the other algorithms; however, the results are telling. The SS-BFE typically selects a much smaller OSB than the SS-MO or the SS-BFE as its main goal is to minimize co-channel interference.

**Figure 27:**
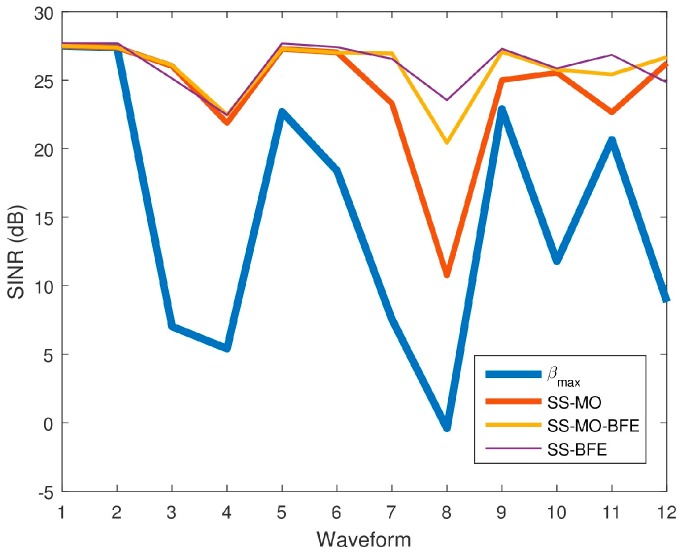
Average signal-to-interference-plus-noise ratio (SINR) values for each SS technique for each pseudo-random waveform.

Figure 27 displays the SINR results for all three algorithms for comparison, while Table 5 reports the SINR performance of each algorithm for the twelve spectra.

Table 6 presents overall performance metrics averaged across all waveforms. The SINR results averaged over all spectra considered reveal that the SINR is highest for the SS-BFE, followed by SS-MO-BFE, SS-MO, and $\beta_{\max}$, in that order. It should be noted, however, that the SINR values produced by each algorithm are relatively close as each algorithm maximizes SINR in its own manner. The principal goal of SS-BFE is to determine an OSB with minimal interference in which a radar can operate and SINR is inherently proportional to the inverse of interference power. Both SS-MO and SS-MO-BFE incorporate an SINR objective function. The significant SINR disparity can be seen between the $\beta_{\max}$ SINR values and the SINR values of the SS-MO, SS-MO-BFE, and SS-BFE OSBs. All three algorithms select an OSB with a considerable SINR improvement over the maximum BW.

### 5.6. Computational Performance

Although the OSB selection algorithms were not designed to optimize computational efficiency, the current computational expense of the algorithms is examined. It should be understood that this computational analysis is not indicative of the maximum attainable efficiency of the algorithm, but rather of the efficiency of each algorithm in its current state of being.

To quantify computational performance, the average processing time for each function is presented. These functions were called consecutively 100 times in a parent function. The input dataset consisted of decimated spectra composed of 2^10^ or 1024 samples. The average computation time for each OSB function is outlined in Table 7. The same processor and analysis platform were used when calculating the average computation time. Processing time is unique to processor speed and analysis platform, therefore the average processing time values presented in Table 7 provide an understanding of the computational expense of each OSB function relative to the other two OSB functions. The least efficient prsssocess among all of the algorithms is computing the brute force PSE matrix. This computation may be able to be streamlined using more efficient matrix math; however, this solution has not been explored.

Another approach was recently investigated to refine the spectral content into a subset of representative information, thereby reducing the number of frequency bins (K) input to SS-MO [14]. This fast SS-MO technique simultaneously reduces the computational complexity while preserving the performance of multi-objective optimization. Future research will investigate the utility of this approach with the proposed OSB selection techniques.

## 6. Conclusions

This paper discusses the SS radar solution space with a focus on statistical spectrum analysis. A SS radar has the same applications and goals of a typical radar system, however it uses knowledge, both *a priori* and active observation, to optimize the radar performance or to increase the radar’s EMC. A statistical framework was established to serve as a foundation for the three OSB selection algorithms that are developed in the paper, namely, the SS-MO, the SS-BFE, and the SS-MO-BFE. These algorithms are designed to determine or define an OSB for radar transmission based on an input spectrum. The SS-MO’s primary goal is to increase radar performance in a congested environment via a WSMO function containing SINR and BW (i.e., range resolution) weighting functions. The SS-BFE’s intention is to utilize the PSE along with Pareto improvement to determine an OSB of a spectrum where a radar will operate while minimizing the co-channel interference it may cause. The SS-MO-BFE technique operates in the middle-ground of the SS-MO and SS-BFE in the sense that its intent is to find a sub-band that allows for increased radar performance and EMC. The SS-MO-BFE uses a WSMO function (much like the SS-MO) that accounts for SINR, range resolution, and PSE weighting functions. All three algorithms were evaluated and compared using ambient and generated data captured by a unique hardware implementation. Depending upon the application, any one of the approaches may be appropriate.

While the paper addresses one specific topic, extensive details that are presented on the hardware and algorithm development will permit others to modify the approach for their own applications. It is imperative that attention be paid to spectrum sensing owing to the rapid spectrum congestion in recent years in order to maintain EMC with and avoid RFI from other users.

Several avenues for improvement are under investigation. The OSB identification techniques have been investigated without considering the hurdles that are associated with real-time implementation. The SAS lays out a hardware that is capable of providing active spectral data; however, real-time processing techniques can be more efficiently implemented. Our analysis is limited to temporally static spectra in order to narrow the problem space. The spectrum, however, is a temporally dynamic entity and must be examined as such. Therefore, a practical OSB technique should incorporate some form of spectrum prediction to account for change over time. As an example, the empirical estimate of entropy defined in Equation (15) does not consider the correlation between sequential samples. The actual entropy, which depends not only on the occurrence frequency of each received signal strength (RSS) level, but also the temporal order in which the RSS levels occurred, can be employed as it captures the full frequency-time structure present in a given spectrum band’s revolution pattern [43].

## Figures and Tables

**Table 1:** Relevant specifications of the analog-to-digital converters (ADCs) and downconverters used in the SAS.

Pentek Card	Type	Band (MHz)	Instantaneous BW (MHz)	Quantity
71741	ADC	0.1–1800	1800	1
71661	ADC	0.1–200	200	8
7120	DC	35–4400	100	4
8111 opt. 1	DC	800–1200	50	1
8111 opt. 2	DC	1100–1500	50	1
8111 opt. 3	DC	1400–1800	50	1
8111 opt. 4	DC	1700–2100	50	1

**Table 2:** Pseudo-random waveforms used in closed loop testing mimicking the ambient data.

Ambient Data			Pseudo-Random Data		
Sub-Band CF (MHz)	Total Avg. Power Range (dBm)	PO Range (%)	Waveform	Total Avg. Power (dBm)	Avg. PO (%)
606	[−77.74, −74.65]	[00.35, 00.93]	1	−74.90	00.21
*1250*	[−77.05, −76.82]	[00.05, 00.09]	2	−74.88	00.06
*300*	[−70.49, −69.80]	[18.81, 21.08]	3	−67.40	14.91
640	[−70.16, −65.84]	[41.88, 46.69]	4	−63.51	31.21
760	[−77.65, −73.06]	[00.00, 03.36]	5	−74.45	02.94
1590	[−74.12, −74.08]	[01.35, 02.10]	6	−73.38	05.52
500	[−75.27, −74.80]	[27.38, 36.97]	7	−67.28	16.40
645	[−72.45, −61.05]	[17.96, 61.19]	8	−58.71	50.07
940	[−76.74, −58.85]	[00.00, 04.36]	9	−74.42	02.50
505	[−77.66, −67.07]	[11.15, 55.70]	10	−69.10	23.45
1800	[−74.20, −74.10]	[01.11, 02.75]	11	−74.03	04.29
1960	[−74.75, −67.28]	[21.56, 49.27]	12	−66.48	16.71

**Table 3:** Average PI performance comparison.

Waveform	Number of Emitters			Avg. PI of Optimal Band of Transmission (%)			
	Sinusoids	Chirps	Total	SS-MO	SS-MO-BFE	SS-BFE	*β*_max_
1	2	0	2	100.00	100.00	15.00	100.00
*2*	1	0	1	10000	100.00	16.00	100.00
*3*	2	0	2	0.00	0.00	0.00	100.00
4	4	1	5	14.00	0.00	0.40	100.00
5	4	0	4	50.00	50.00	7.00	100.00
6	4	0	4	50.00	50.00	12.50	100.00
7	3	0	3	67.50	25.00	0.00	100.00
8	2	1	3	62.67	0.00	0.00	100.00
9	3	0	3	66.67	33.33	14.00	100.00
10	5	1	6	38.67	33.33	0.33	100.00
11	4	1	5	82.00	27.20	13.20	100.00
12	3	0	3	10.00	0.00	0.00	100.00

**Table 4:** OSB BW performance comparison.

Waveform	Avg. BW of Optimal Band of Transmission (MHz)			
	SS-MO	SS-MO-BFE	SS-BFE	*β*_max_
1	35.00	35.00	19.35	35.00
*2*	35.00	34.99	19.84	35.00
*3*	15.52	15.35	12.14	35.00
4	14.06	10.51	10.22	35.00
5	31.24	31.24	14.70	35.00
6	28.59	28.65	17.55	35.00
7	25.01	12.00	11.70	35.00
8	31.78	11.32	7.94	35.00
9	30.85	22.50	14.09	35.00
10	15.90	14.50	5.79	35.00
11	31.40	18.82	10.87	35.00
12	22.57	21.23	13.47	35.00

**Table 5:** SINR performance comparison.

Waveform	Avg. SINR of Optimal Band of Transmission (dB)			
	SS-MO	SS-MO-BFE	SS-BFE	*β*_max_
1	27.49	27.49	27.70	27.49
*2*	27.37	27.37	27.70	27.37
*3*	26.03	26.08	25.12	7.02
4	21.86	22.46	22.45	5.40
5	27.27	27.27	27.67	22.69
6	27.03	27.01	27.40	18.39
7	23.29	26.95	26.54	7.55
8	10.73	20.43	23.54	−0.41
9	24.99	27.06	27.29	22.90
10	25.54	25.75	25.85	11.77
11	22.65	25.42	26.84	20.65
12	26.23	26.67	24.82	8.83

**Table 6:** Comparison of overall performance metrics averaged across all waveforms.

Metric	SS-MO	SS-MO-BFE	SS-BFE	*β*_max_
SINR (dB)	24.21	25.83	26.08	14.97
β (MHz)	26.41	21.34	13.14	35
PI (%)	53.46	34.91	8.33	100

**Table 7:** Processing time of each OSB selection technique for averaged over 100 decimated spectra with 2^10^ samples.

OSB Algorithm	Processing Time (ms)
SS-MO	195.57
SS-MO-BFE	5120.74
SS-BFE	4995.84

## References

[1] Zhao Q., Sadler B.M. (2007). A survey of dynamic spectrum access. IEEE Signal Process. Mag..

[2] Federal Communications Commission Spectrum Policy Task Force (2002). Report of the Spectrum Efficiency Working Group.

[3] Sherman M., Mody A.N., Martinez R., Rodriguez C., Reddy R. (2008). IEEE standards supporting cognitive radio and networks, dynamic spectrum access, and coexistence. IEEE Commun. Mag..

[4] Zhao Q., Swami A. A survey of dynamic spectrum access: signal processing and networking perspectives. Proceedings of the IEEE International Conference on Acoustics, Speech and Signal Processing (ICASSP 2007).

[5] Jayaweera S.K., Li T. (2009). Dynamic spectrum leasing in cognitive radio networks via primary-secondary user power control games. IEEE Trans. Wirel. Commun..

[6] Kundargi N., Tewfik A. Hierarchical sequential detection in the context of dynamic spectrum access for cognitive radios. Proceedings of the 14th IEEE International Conference on Electronics, Circuits and Systems (ICECS 2007).

[7] Haykin S. (2006). Cognitive radar: a way of the future. IEEE Signal Process. Mag..

[8] Guerci J.R. Cognitive radar: a knowledge-aided fully adaptive approach. Proceedings of the 2010 IEEE Radar Conference.

[9] Martone A.F. (2014). Cognitive radar demystified. URSI Radio Sci. Bull..

[10] Haykin S., Thomson D.J., Reed J.R. (2009). Spectrum sensing for cognitive radio. Proc. IEEE.

[11] Yücek T., Arslan H. (2009). A survey of spectrum sensing algorithms for cognitive radio applications. IEEE Commun. Surv. Tutor..

[12] Martone A., Sherbondy K., Ranney R., Dogaru T. Passive sensing for adaptable radar bandwidth. Proceedings of the 2015 IEEE Radar Conference.

[13] Stinco P., Greco M.S., Gini F. (2016). Spectrum sensing and sharing for cognitive radars. IET Radar Sonar Navig..

[14] Martone A., Ranney K., Sherbondy K., Gallagher K., Blunt S. (2018). Spectrum allocation for non-cooperative radar coexistence. IEEE Trans. Aerosp. Electron. Syst..

[15] Romero R.A., Goodman N.A. (2009). Waveform design in signal-dependent interference and application to target recognition with multiple transmissions. IET Radar Sonar Navig..

[16] Aubry A., De Maio A., Huang Y., Piezzo M., Farina A. (2015). A new radar waveform design algorithm with improved feasibility for spectral coexistence. IEEE Trans. Aerosp. Electron. Syst..

[17] Huang K.-W., Bică M., Mitra U., Koivunen V. Radar waveform design in spectrum sharing environment: Coexistence and cognition. Proceedings of the 2015 IEEE Radar Conference.

[18] Kirk B.H., Narayanan R.M., Martone A.F., Sherbondy K.D. Waveform design for cognitive radar: target detection in heavy clutter. Proceedings of the SPIE Conference on Radar Sensor Technology XX.

[19] Kirk B.H., Owen J.W., Narayanan R.M., Blunt S.D., Martone A.F., Sherbondy K.D. Cognitive software defined radar: waveform design for clutter and interference suppression. Proceedings of the SPIE Conference on Radar Sensor Technology XXI.

[20] Pooler R.K., Narayanan R.M., Sherbondy K.D., Martone A.F., Gallagher K.A. A dynamic spectrum analysis solution for the characterization of the UHF spectrum. Proceedings of the SPIE Conference on Radar Sensor Technology XX.

[21] Martone A., Gallagher K., Sherbondy K., Hedden A., Dietlein C. (2017). Adaptable waveform design for enhanced detection of moving targets. IET Radar Sonar Navig..

[22] Chiang R.I.C., Rowe G.B., Sowerby K.W. A quantitative analysis of spectral occupancy measurements for cognitive radio. Proceedings of the 65th IEEE Vehicular Technology Conference (VTC2007-Spring).

[23] Mehdawi M., Riley N., Paulson K., Fanan A., Ammar M. (2013). Spectrum occupancy survey in HULL-UK for cognitive radio applications: measurement & analysis. Int. J. Sci. Technol. Res..

[24] López-Benítez M., Umbert A., Casadevall F. Evaluation of spectrum occupancy in Spain for cognitive radio applications. Proceedings of the 69th IEEE Vehicular Technology Conference (VTC2009-Spring).

[25] Spaulding A., Hagn G.H. (1977). On the definition and estimation of spectrum occupancy. IEEE Trans. Electromagn. Compat..

[26] Wellens M., de Baynast A., Mahonen P. (2008). Performance of dynamic spectrum access based on spectrum occupancy statistics. IET Commun..

[27] Roberson D.A., Hood C.S., LoCicero J.L., MacDonald J.T. Spectral occupancy and interference studies in support of cognitive radio technology deployment. Proceedings of the 1st IEEE Workshop on Networking Technologies for Software Defined Radio Networks (SDR ’06).

[28] Lathi B.P. (1998). Signal Processing and Linear Systems.

[29] Srinu S., Sabat S.L. (2012). FPGA implementation and performance study of spectrum sensing based on entropy estimation using cyclic features. Comput. Electr. Eng..

[30] Zhang Y.L., Zhang Q.Y., Melodia T. (2010). A frequency-domain entropy-based detector for robust spectrum sensing in cognitive radio networks. IEEE Commun. Lett..

[31] Wehner D.R. (1987). High Resolution Radar.

[32] Keep D.N. (1956). Frequency-modulation radar for use in the mercantile marine. Proc. Inst. Elect. Eng. Part B Radio Electron. Eng..

[33] Rogers D.G. (1978). Pascal triangles, Catalan numbers and renewal arrays. Discrete Math..

[34] Smith K.A. A genetic algorithm for the channel assignment problem. Proceedings of the IEEE Global Telecommunications Conference (GLOBECOM 1998).

[35] Cohen B. Incentives build robustness in BitTorrent. Proceedings of the Workshop on Economics of Peer-to-Peer Systems.

[36] Abbass H.A., Sarker R., Newton C. PDE: A Pareto-frontier differential evolution approach for multi-objective optimization problems. Proceedings of the 2001 IEEE Congress on Evolutionary Computation.

[37] Szeliski R. (2006). Image alignment and stitching: A tutorial. Found. Trends Comput. Graphics Vision.

[38] Shi J., Tomasi C. Good features to track. Proceedings of the 1994 IEEE Computer Society Conference on Computer Vision and Pattern Recognition (CVPR ‘94).

[39] Harris C., Stephens M. A combined corner and edge detector. Proceedings of the Fourth Alvey Vision Conference.

[40] Pooler R.K. (May 2017). An Analysis of Spectrum Sharing for Radar Applications. M.S. Thesis.

[41] Li W., Zhang Y., Lin J., Chen Z. IF digitization receiver of wideband digital array radar test-bed. Proceedings of the SPIE Conference on Millimetre Wave and Terahertz Sensors and Technology VII.

[42] Sanders F.H., Sole R.L., Carroll J.E., Secrest G.S., Allmon T.L. (2012). Analysis and Resolution of RF Interference to Radars Operating in the Band 2700–2900 MHz from Broadband Communication Transmitters.

[43] Ding G., Wang J., Wu Q., Yao Y.-D., Li R., Zhang H., Zou Y. (2015). On the limits of predictability in real-world radio spectrum state dynamics: From entropy theory to 5G spectrum sharing. IEEE Commun. Mag..

